# Prevalence of Hepatitis C Virus Genotypes in District Bannu, Khyber Pakhtunkhwa, Pakistan

**DOI:** 10.1155/2014/165826

**Published:** 2014-06-30

**Authors:** Shamim Saleha, Anwar Kamal, Farman Ullah, Nasar Khan, Asif Mahmood, Sanaullah Khan

**Affiliations:** ^1^Department of Microbiology, Kohat University of Science and Technology (KUST), Khyber Pakhtunkhwa, Kohat 26000, Pakistan; ^2^Department of Biotechnology and Genetic Engineering, Kohat University of Science and Technology (KUST), Khyber Pakhtunkhwa, Kohat 26000, Pakistan; ^3^Department of Zoology, Kohat University of Science and Technology (KUST), Khyber Pakhtunkhwa, Kohat 26000, Pakistan

## Abstract

Determination of an individual's hepatitis C virus (HCV) genotypes prior to antiviral therapy has become increasingly important for the clinical management and prognosis of HCV infection. Therefore, this study was conducted to investigate the prevalence of HCV genotypes in HCV infected patients of district Bannu in Khyber Pakhtunkhwa region of Pakistan. Serum samples of 117 seropositive patients were screened for HCV-RNA by using reverse transcriptase-nested polymerase chain reaction (RT-nested PCR) and then PCR positive samples were subjected to HCV genotyping. Out of 117 seropositive samples, 110 samples were found positive by PCR analysis. Genotype 3a was the most prevalent one detected in 38% of patients, followed by genotype 3b in 21% of patients, and then genotype 2a in 12% of patients. However 21% of HCV-PCR positive samples could not be genotyped by method used in this study. Genotype 3a was the most prevalent genotype in patients of all age groups and its prevalence was found high among patients with increasing age (>34 years). Moreover, genotypes 3a and 3b were found to be the most prevalent genotypes in patients with history of shaving by barbers, receiving multiple injections, and dental procedures. In conclusion there is need of further investigation of genotypes of HCV by using more sensitive assays and considering large sample size in district Bannu.

## 1. Introduction

HCV infection is among life threatening public health problems worldwide, with over 170–200 million infected people [1] including about 17 million from Pakistan [2]. HCV is considered the leading cause of liver cirrhosis and hepatocellular carcinoma. It has been estimated to cause approximately 27% of cirrhosis and 25% of hepatocellular carcinoma cases worldwide [3]. Each year about 350,000 people die due to HCV [4].

HCV is a small enveloped, positive sense single stranded RNA virus and has been classified as a separate genus* hepacivirus* in the Flaviviridae family [5]. The HCV genome is approximately 9.6 kb, encoding a polyprotein of about 3010 amino acids and is flanked by short untranslated regions (UTRs) regions at the 5′ and 3′ terminus [6]. This polyprotein is posttranslationally processed by viral and cellular proteins to generate the structural proteins (C, E1, E2, and p7) and nonstructural proteins (NS2, NS3, NS4A, NS4B, NS5A, and NS5B), [7].

HCV shows high degree of genetic heterogeneity; consequently six major genotypes and multiple subtypes of HCV have been identified so far in world [8]. Distribution of HCV genotypes and subtypes in different regions of the world is variable. The common subtypes found in North and South America, Europe, Russia, China, Japan, Australia, and New Zealand are 1a, 1b, 2a, 2c, and 3a [3]. Genotype 4 is predominant in Egypt, North Africa, Central Africa, and Middle East [9]. Genotype 5 in South Africa [10] and genotype 6 in Southeast Asia [11] have been identified. Genotype 3 is the most prevalent genotype in India, Bangladesh, Pakistan, and Nepal [12–15]. Subtypes of genotypes 1, 2, 3, and 6 have been found prevalent in Thailand [16], Vietnam, Indonesia, and Burma [1] respectively. Genotype 1b is the most prevalent genotype in China; however genotype 2 has also been reported from some regions of China [1].

In Pakistan, the prevalence of HCV infection has been estimated to be 8% and is increasing gradually due to deficiency in basic health care recourses and lack of the general public awareness about safety measures [8]. Some studies have been conducted on prevalence of HCV genotypes in Khyber Pukhtunkhwa region of Pakistan [17–20]. However, little has been reported on prevalence of HCV genotypes in district Bannu in Khyber Pukhtunkhwa region of Pakistan. Therefore, this study was conducted to find out baseline information on the prevalence of HCV genotypes in district Bannu. Accurate HCV genotyping can be used in better understanding of HCV infection, for creating awareness in the general public and subsequently for implementation of preventive and therapeutic strategies.

## 2. Materials and Methods

### 2.1. Ethical Consideration

All the procedures used in this study were approved by the Ethics Committees of Department of Microbiology, Kohat University of Science and Technology. The informed consent was signed by the patients for participating in the study.

### 2.2. Inclusion Criteria

An inclusion criterion for patients was to be seropositive for anti-HCV by third generation enzyme linked immunosorbent assay (ELISA). The information regarding age, gender, and possible routes of transmission was obtained from each participating patient. Total 117 blood samples were collected from patients attending district hospital Bannu and Khalifa hospital Bannu.

### 2.3. DNA Extraction

Serum was separated from each blood sample at 3000 ×g for 5 min and then labeled and stored deep-frozen at −20°C. RNA was extracted using RNA extraction Kit (Ultrascript, Anagen Technologies Inc., USA) as per manufacturer's instructions.

### 2.4. Genotyping

Extracted RNA was reverse transcribed into complementary DNA (cDNA). For this 10 *μ*L of HCV extracted RNA was incubated at 37°C for 50 min along with primer specific for core region and 200 U of Moloney Murine Leukemia Virus reverse transcriptase (M-MLV RTase) (Fermentas USA), 5X first strand buffer (MMulv buffer) dNTPs and ddH_2_O. In the first round of Nested PCR, cDNA was amplified by using sense and antisense primers for qualitative analysis. The PCR program was as follows: initial denaturation was at 94°C for 5 min, followed by 45 cycles, each of 45 sec denaturation at 92°C, 45 sec annealing at 55°C, and 1 min extension at 72°C, with final extension at 72°C for 10 min. In second round of Nested PCR, genotype-specific PCR was performed by using allele specific primers for core region reported by Ohno et al. [21] at same PCR program that was adopted for first round Nested PCR. The final PCR products obtained after each round of Nested PCR were subjected to electrophoresis and separated on 2% agarose gel. After staining with ethidium bromide, the gel was visualized under UV-transilluminator. To determine genotype specific bands, the banding pattern was photographed in Gel Documentation System (ENDURO GDS).

## 3. Results

**Figure 1:**
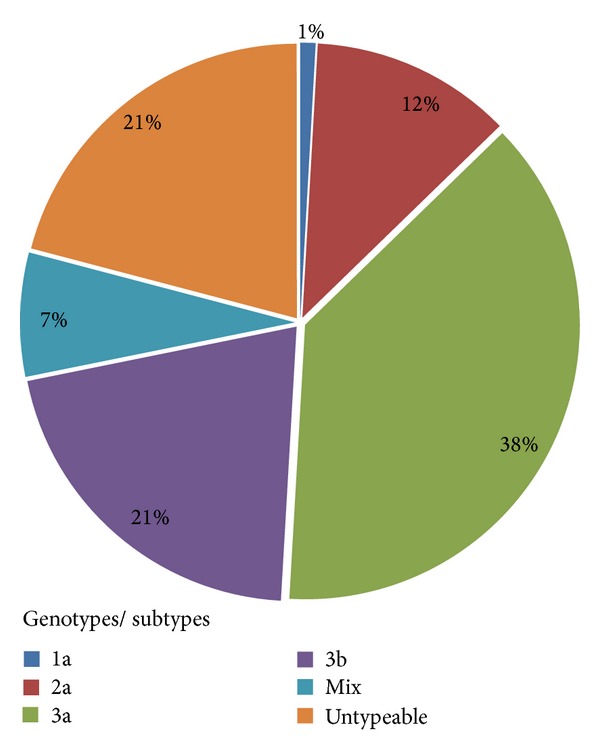
Prevalence of HCV genotypes in district Bannu.

Out of 117 anti-HCV positive sera by ELISA, 110 samples were found positive by PCR analysis, with greater representation of males 81 (73.6%) as compared to females 29 (26.4%) as shown in Table 1. Genotyping of 110 HCV-PCR positive samples determined four different genotypes including 1a, 2a, 3a, and 3b (Figure 1). However, HCV genotypes 1b, 2b, 4, 5, and 6 were not detected among patients studied. Genotype 3a was the most prevalent one detected in 42 (38%) patients, followed by genotype 3b in 23 (21%) patients and then genotype 2a in 13 (12%) patients. Genotype 1a was the least prevalent and was detected in only in 1 (1%) patient while in 8 (7%) patients mixed genotypes of HCV were detected. Moreover, 23 (21%) HCV-PCR positive samples could not be genotyped by using the method as described previously [21].

The studied patients were categorized in three different age groups and then prevalence of age associated HCV genotypes was determined (Table 1). Genotype 3a was most prevalent genotype in all age groups patients and its prevalence was found high among patients with increasing age (>34 years). High prevalence of genotype 3b was observed in age groups 35–54 and 55–74 years. Similarly, high prevalence of genotype 2a was also observed in age group 55–74 years. Moreover, genotype 1a was found in only one patient, who had age of 23 years and subsequently belonged to age group 15–34 years.

In our study, those patients who had history of visit to barber shop, intravenous drug addiction, and dental procedures were recorded major risk factors responsible for HCV transmission in district Bannu as shown in Table 2. Patients with history of visit to barber shop and receiving multiple therapeutic injections accounted for 40 (36.4%) and 39 (35.4%), respectively, followed by patients with history of dental procedures 22 (20%). History of blood transfusion and tattooing was recorded in 7 (6.4%) and 2 (1.8%) patients, respectively.

Genotype 3a and genotype 3b were found to be the most prevalent genotypes in patients with history of shaving by barbers, receiving multiple injections, and dental procedures. However, genotype 2a and genotype 1a were more commonly found in patients with history of dental procedures (Table 2).

## 4. Discussion

The molecular epidemiological studies have reported that significant regional differences appear to be present in the frequency distribution of HCV genotypes. Moreover, determination of HCV genotypes in geographically diverse regions facilitates therapeutic decisions and preventive strategies [22]. It has been reported that there are variations in disease outcome and response to antiviral therapy of HCV genotypes [23]. However, in Pakistan treatment of HCV infected patients is based on qualitative or quantitative viral detection and genotypes are not determined prior to treatment. Therefore variable response rates of HCV infected patients to antiviral therapy cannot be detected. The present study was conducted to determine baseline data on the prevalence of HCV genotypes in a district in Khyber Pakhtunkhwa region of Pakistan. The baseline information will help in better understanding of HCV infection, awareness in the general public and subsequent control strategies.

The distribution of HCV genotypes was found variable among studied patients. The genotype 3a was found to be the most prevalent genotype followed by 3b and 2a and genotype 1a was found to be less prevalent (Figure 1). Results of the present study are in conformity with results of previous studies reported from different regions of the Khyber Pakhtunkhwa in Pakistan [20–25]. Previous studies conducted in India, Bangladesh, and Nepal also reported that the genotype 3 is the most prevalent genotype [12–14]. In this study the assay used could not determine HCV genotypes among a considerable number of HCV patients (23%). Untypeable genotypes have previously been reported in another study conducted in Pakistan [25]. However, there is a need to use more reliable and sensitive assay for genotyping of HCV in untypeable samples.

The distribution of HCV genotypes may be variable among the patients of different age groups. Studies have reported that genotypes 1b and 2 were more prevalent in older patients, whereas genotype 1a was observed more frequently in the younger population [26, 27]. Another study reported that in France genotype 5 was frequently detected in patients aged more than 50 years [28]. In Iran genotype 3a was the most frequently detected in patients less than 40 years [29]. In this study, we observed the distribution of HCV genotypes among different age groups. The prevalence of genotypes 2a and 3a was found increasing with increasing age of patients. Genotype 3b was found more prevalent in age groups more than 35 years. Moreover, genotype 1a was least prevalent genotype detected in a patient of a younger age group less than 34 years. The findings of this study are important for therapeutic management of HCV infected patients.

Various studies have suggested that HCV genotypes are associated with different routes of transmission. Analysis of possible routes in transmission of HCV genotypes in district Bannu is shown in Table 2. The HCV genotypes reported in present study were isolated from participating patients with known route of transmission. In our study genotypes 3a and 3b were more frequently observed in patients with previous history of shaving by barbers followed by multiple injections received and dental procedures. Whereas genotypes 1a and 2a were more commonly observed in patients who had history of dental procedures. HCV genotype 2a was also common in patients with previous history of visit to barbers and receiving multiple injections.

The possible routes of transmission of HCV genotypes have also been reported in other studies. The high prevalence of HCV genotype 3 is attributed to intravenous drug addicts in the United States and Europe [30]. Moreover present study and other studies from Pakistan have also reported increased prevalence of genotype 3 in those patients who had received multiple therapeutic unsafe and unnecessary injections by untrained health practitioners particularly in rural areas. These untrained health practitioners usually use nondisposable syringe or used syringe and needles for more than one patient at the public health-care centers [24–32]. However, high prevalence of genotypes 3a, 3b, and 2a among patients of district Bannu with history of shaving by barbers and dental procedures has not been reported in the United States and Europe. In present study we observed in district Bannu that uneducated barbers common practice is to reuse of unsterilized razors and scissors for multiple customers. Similarly untrained health practitioners at dental clinics are usually in practice of using used and unsterilized dental equipment for multiple individuals. Consequently, these barbers and health practitioners are promoting the risk of transmission of HCV infection from one person to another in this district. In a patient with genotype 1a the possible route of transmission observed in the present study was dental procedure. This is consistent with result of a previous study from Pakistan where most of patients with genotype 1a had a history of dental procedures [24].

## 5. Conclusion

Our study provides baseline information on the prevalence of HCV genotypes in district Bannu in Khyber Pukhtunkhwa region of Pakistan. The most prevalent HCV genotype was 3a isolated from patients in district Bannu, followed by genotypes 3b and 2a. The frequency distribution of these genotypes was found variable according to the age groups of the patients studied. The possible routes of transmission for these genotypes observed were shaving by barbers, receiving multiple injections, and dental procedures. Further studies are needed to investigate HCV genotypes in district Bannu by using more sensitive assays and considering large population size.

## Figures and Tables

**Table 1:** Prevalence of HCV genotypes among the patients of different age groups.

Age groups	Genotypes						Total	% age
	1a	2a	3a	3b	Mix	Untypeable		
15–34	1	1	7	5	4	3	21	19.1
35–54	0	3	17	9	3	12	44	40
55–74	0	9	18	9	1	8	45	40.9

Total	1	13	42	23	8	23	110	100

**Table 2:** Possible routes of transmission of HCV genotypes among the patients.

Possible routes of transmission	Genotypes						Total	% age
	1a	2a	3a	3b	Mix	Untypeable		
Multiple therapeutic injections received	0	3	19	10	1	6	39	35.4
Dental procedures	1	5	6	5	2	3	22	20
Blood transfusion	0	1	2	0	1	3	7	6.4
Shaving by barber	0	4	15	7	4	10	40	36.4
Tattooing	0	0	0	1	0	1	2	1.8

Total	1	13	42	23	8	23	110	100
